# Etiologies of Multidrug-Resistant Epilepsy in Latin America: A Comprehensive Review of Structural, Genetic, Metabolic, Inflammatory, and Infectious Origins: A Systematic Review

**DOI:** 10.3390/biom15040576

**Published:** 2025-04-12

**Authors:** Mario S. Hinojosa-Figueroa, Mishell Cruz-Caraguay, Alejandro Torres Pasquel, Vanesa Puga Rosero, Camila Belen Eguiguren Chavez, Jose A. Rodas, Jose E. Leon-Rojas

**Affiliations:** 1 NeurALL Research Group, Quito 170157, Ecuador; mariohinojosaf.0711@gmail.com (M.S.H.-F.); macruz29@utpl.edu.ec (M.C.-C.); altorrespa@uide.edu.ec (A.T.P.); vapugaro@uide.edu.ec (V.P.R.); cbeguigurenc@gmail.com (C.B.E.C.); 2 Escuela de Medicina, Universidad Internacional del Ecuador, Quito 170411, Ecuador; 3 Facultad de Ciencias de la Salud, Universidad Técnica Particular de Loja, Loja 1101608, Ecuador; 4 School of Psychology, University College Dublin, D04 C1P1 Dublin, Ireland; 5 Escuela de Psicología, Universidad Espíritu Santo, Samborondón 092301, Ecuador; 6 Escuela de Medicina, Universidad de las Américas, Quito 170124, Ecuador

**Keywords:** multidrug-resistant epilepsy, prevalence, causes, etiology, Latin America

## Abstract

Epilepsy is a prevalent neurological disorder that affects millions worldwide, with a significant portion of individuals experiencing drug-resistant forms of the condition. In Latin America, the challenge of identifying the underlying causes of multidrug-resistant epilepsy (MDRE) is particularly pressing. (1) Background: This systematic review aims to highlight the critical importance of understanding the etiology of MDRE in Latin America. (2) Methods: A systematic review of Medline (PubMed), Scopus, and Web of Science was conducted following the PRISMA methodology; articles were selected if they included information on the etiology of MDRE in Latin-American participants, and the NHLBI tool was used to assess bias. (3) Results: A total of 37 published articles were finally included in the review. The most frequently documented cause of drug-resistant epilepsy was structural, affecting 725 patients, with hippocampal atrophy and sclerosis predominantly involving both the right and left lobes. The second most common cause was genetic, identified in 362 individuals who exhibited polymorphisms in genes such as *ABCB1*, *CYP2C9*, *SCN1A*, *SLC6A4*, and *MDR-1*, among others. The third most frequent cause was metabolic, and the fourth was inflammatory, affecting 258 individuals, which was associated with various inflammatory markers, including IL-1β, IL-6, CD8+, CD-25, and HLA-DR. Finally, infectious causes were also reported. (4) Conclusions: Structural causes are the leading etiology of MDRE in Latin America, followed by genetic, metabolic, inflammatory, and infectious origins. The regional pattern contrasts with findings from Europe and Asia, highlighting the influence of socioeconomic, environmental, and population-specific genetic factors. Our findings underscore the urgent need for regionally tailored research and interventions, particularly in understudied areas such as infectious causes and neuroinflammation.

## 1. Introduction

Epilepsy is a serious neurological condition that impacts approximately 70 million individuals globally; this condition not only increases the risk of morbidity and mortality but also significantly diminishes the quality of life for those affected [1]. While anti-seizure medication (ASM) therapy effectively controls seizures in the majority, about 30–40% of epilepsy patients do not respond to these treatments; this is a condition known as multidrug-resistant epilepsy (MDRE). Those with MDRE face substantial socioeconomic and psychological challenges that further reduce their quality of life and heighten their risk of mortality [2]. MDRE is defined by the International League Against Epilepsy (ILAE) as a lack of seizure freedom despite adequate trials of at least two or more regimens of ASM [1].

Studying MDRE is crucial, particularly in Latin America, where there is a notable lack of comprehensive databases on both etiology and potential treatments, as well as an underestimation of diagnosis; despite this, the prevalence of MDRE has been reported to be approximately 30% [3]. For example, the parasitic infection caused by Taenia solium—cysticercosis—and its neurological syndrome (neurocysticercosis) are endemic to Latin-American countries. This infection is associated with MDRE, and even though in the last few decades the reported cases have lowered in countries like Brazil, Ecuador, and Mexico, there have also been significant increases in other countries, such as Colombia [4]. Because of this, it is imperative to investigate the causes of MDRE (including its infectious etiologies) [4].

The scarcity of detailed and accessible information hampers the ability to accurately diagnose and effectively manage this complex condition; therefore, by focusing research efforts on MDRE, we can address these gaps in understanding, develop targeted therapies, and ultimately improve patient outcomes [3]. Here, we provide an updated compendium of the most prevalent causes of refractory epilepsy in Latin America and the importance of these in patient diagnosis and treatment.

## 2. Materials and Methods

The following systematic review was written in accordance with the Preferred Reporting Items for Systematic Reviews and Meta-Analysis (PRISMA) 2020 guideline, and the protocol was registered in PROSPERO (CRD 42022377248).

### 2.1. Eligibility Criteria

The inclusion criteria used for the articles included primary studies (cross-sectional, cohort, case–control studies, and other observational studies) that included Latin-American participants diagnosed with MDRE; there were no date, sex, or cultural context restrictions. The exclusion criteria included secondary studies (systematic and literary reviews), letters to the editor, animal experimentation, or in vitro studies. Patients with convulsive crisis without a clear diagnosis, patients with non-refractory epilepsy only, and patients without a clear etiology were also excluded.

### 2.2. Language

Articles in both English and Spanish were included. Spanish was a necessary inclusion criterion as our review focuses on MDRE in Latin-American countries.

### 2.3. Information Sources

We queried Medline (PubMed), Scopus, and Web of Science (WOS) from inception until 22 November 2022. No filters were applied to any databases.

### 2.4. Search Strategy

The specific search protocols and keywords used in each database are provided in the Supplementary Files. We conducted an independent search and screening of the articles; in cases of disagreement, we conducted a thorough discussion until we reached a mutual consensus. A total of 1425 articles were retrieved using PubMed, Scopus, and WOS; each article was assessed by two blinded reviewers, first analyzing the abstract, title, and keywords, and at a later stage, analyzing the full text against the aforementioned inclusion and exclusion criteria.

### 2.5. Data Management

All articles extracted from the databases were imported into Ryyan (a web-based systematic review program) for screening and duplicate resolution; this aided in minimizing bias and data entry errors.

### 2.6. Selection Process

The aforementioned eligibility criteria were used to screen all titles, abstracts, and keywords; following this first phase, articles that satisfied the inclusion criteria underwent a thorough full-text review. Ryyan was used, independently and in a blinded fashion, by two reviewers during the complete selection process and conflict resolution.

### 2.7. Data Items

After the selection process, data from the articles were extracted, compiled, and organized in a Microsoft Excel (Microsoft Corporation, Redmond, USA) spreadsheet; extracted information included article type, number of participants, country, method of diagnosis, type of epilepsy, time of resistance to treatment, drugs previously used, identified cause of refractory epilepsy, and results with statistical tests and analyses.

### 2.8. Bias Assessment

We used the National Heart, Lung, and Blood Institute’s (NHLBI) study quality assessment tools for bias assessment. These tools comprised a set of questionnaires tailored to the specific type of study design being evaluated, including case–control studies, controlled intervention studies, cross-sectional studies, and observational cohort studies. The degree of bias was categorized as low, moderate, or high based on the percentage of affirmative responses to the questions posed. A low risk of bias was indicated if 80% or more of the questions were answered affirmatively, while a moderate risk of bias was indicated if the percentage of affirmative responses was between 50% and 79%; finally, a high risk of bias was assigned if less than 50% of the questions were answered affirmatively.

### 2.9. Effect Measures and Synthesis Methods

Our study aimed to characterize the etiology of MDRE in Latin-American patients through a qualitative systematic review reporting absolute and relative frequencies as our outcome measures; therefore, a meta-analysis was not feasible because of the significant heterogeneity that can occur due to the inclusion of different etiologies and diagnostic criteria. Our included studies were tabulated based on the type of MDRE and the etiology reported (structural, metabolic, genetic, inflammatory, or infectious); only MDRE participants with a clear influence or association with the potential causality were included for the final tabulation and calculation of the relative frequencies.

## 3. Results

### 3.1. Study Selection

**Figure 1:**
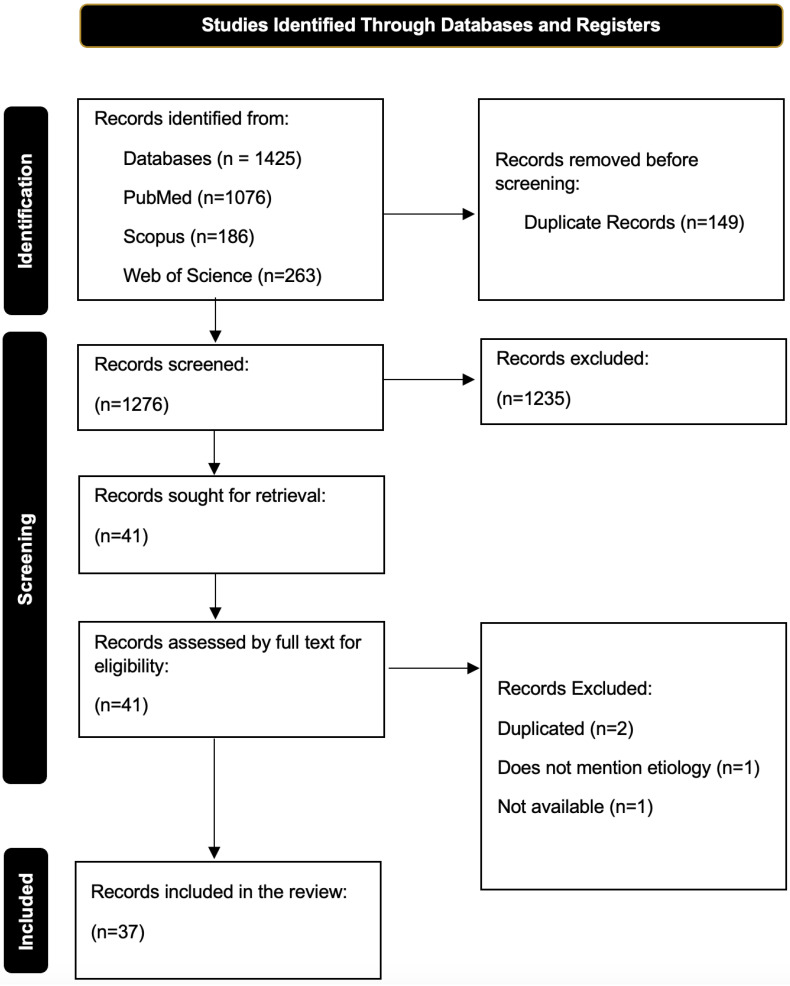
PRISMA flowchart of the selection process.

A total of 1425 articles were found, of which 149 articles were duplicates and 137 were subjected to full-text analysis; of these, 37 articles were finally selected for our review [5,6,7,8,9,10,11,12,13,14,15,16,17,18,19,20,21,22,23,24,25,26,27,28,29,30,31,32,33,34,35,36,37,38,39,40,41]. The complete selection process can be found in Figure 1.

### 3.2. Risk of Bias

The results of the risk of bias analyses of each study can be found in Table 1.

**Figure 2:**
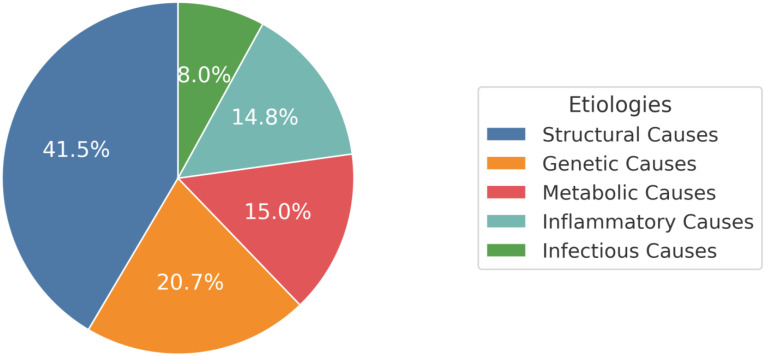
Pie chart showcasing the distribution of the relative frequencies of MDRE etiologies in Latin America.

A total of 3725 patients were included in this review, of which 1746 (46.9%) had an MDRE diagnosis, and were considered to determine the frequency of the different etiologies; the remainder were either control participants or patients with MDRE whose diagnoses were proven to not be genetic (i.e., they had no gene mutations or polymorphisms related to their MDRE). Figure 2 showcases the distribution of the relative frequencies of MDRE etiologies in Latin America.

### 3.3. Structural Causes

In Latin America, structural causes of epilepsy were the most frequently reported in our review, representing 41.5% (n = 725) of the MDRE patients. The most frequent structural defect was hippocampal atrophy/sclerosis affecting 51.7% (n = 375), followed by non-specific structural abnormalities affecting 13.8% (n = 100), traumatic brain injury/strokes affecting 10.5% (n = 76), cortical dysplasia affecting 9.1% (n = 66), gliosis affecting 5.4% (n = 39), vascular malformations affecting 5.1% (n = 37), and central nervous system tumors affecting 4.4% (n = 32) [5,6,7,8,9,10,11,12,13,14,15,16].

Hippocampal atrophy/sclerosis was present in the majority with the involvement of the right and left temporal lobes and, in certain cases, bilaterally, assessed by volumetric and structural magnetic resonance imaging (MRI) [5,6,7,8,9,10,11,12,13,14,15,16]. The gray matter of the hippocampus, anterior cingulate cortex, amygdala, and thalami was shown to be decreased in these patients [5,6,7,8,9,10,11,12,13]. With regards to cortical dysplasia, most articles did not point toward the predominant involvement of a specific lobe; conversely, reactive gliosis was mostly found in the temporal lobe with the direct involvement of the hippocampus [14,15,16]. Vascular malformations, tumors, traumatic brain injuries, and cerebral infarctions were not as frequent; therefore, we did not have enough information to determine specific lobar involvement for the genesis of MDRE [6,13].

### 3.4. Genetic Causes

The multiple genes are associated with the pathogenesis of MDRE; however, some specific gene mutations are more frequently present in patients of Latin-American descent. These genetic alterations are associated with MDRE or with its response to ASM in 20.7% (n = 362) of patients in our review [21,22,23,24,25,26,27,28,29,30,31,32,33,34,35].

Some genes have been reported to increase the risk of MDRE; for instance, the genes of the ATP Binding Cassette (*ABC*), a transmembrane protein involved in interactions with ASMs, have been found to be associated with MDRE. However, in our review, Latin-American patients seemed to not experience this increased risk of MDRE when presenting polymorphisms and/or alterations in this and other genes [21,22]. For example, two studies in Colombia found that polymorphisms and chromosomic aberrations were not associated with MDRE [21,24]. The first study showed that polymorphisms in the *ABCB1, CYP2C9*, or *SCN1A* genes were not associated with a lack of response to phenytoin and that a decreased function in the alleles of *CYP2C9* was associated with more cerebellar and vestibular adverse effects than ASMs [21]. The second study found genetic alterations in only 0.9% of pediatric patients diagnosed with MDRE due to a duplication in chromosomic region 3p26.3, related to exon 3 of the *CHL1* gene [24]. In a similar fashion, a Peruvian study showed a lack of association between polymorphisms in the *ABCB1* (variant *C3435T*) and *ABCC2* (variant *-24C>T*) genes and pharmacoresistance to multiple ASMs like carbamazepine, valproic acid, phenytoin, lamotrigine, and others [22].

In contrast, a study looking at Mexican MDRE pediatric patients found that single-nucleotide polymorphisms (SNPs) in cytochrome P450 (CYP450), an important metabolizer of ASMs, were associated with MDRE in this population, specifically, CYP2D6*2 (rs16947), CYP2D6*4 (rs1065852), CYP2C19*2 (rs4244285), and CYP3A4*1B (rs2740574) [23]. In addition, three Mexican studies looking at 64 MDR temporal lobe epilepsy (TLE) patients analyzed the resected hippocampi looking for alterations in the expression of different genes coding for factors or receptors [29,30,31]. The first study looked at brain-derived neurotrophic factor (BDNF) expression by examining its exon transcripts; they found an increase in the expression of BDNF’s exon VI within the resected epileptic tissue (*p* < 0.05) and no changes in DNA methylation [29]. The second study looked at the Repressor Element-1 Silencing Transcription Factor or Neuron-Restrictive Silencer Factor (*REST/NRSF*), a zinc finger transcription blocker, and showed that patients with MDRE had increased *REST/NRSF* mRNA and protein expression in the resected hippocampi (146% and 273%, respectively; *p* < 0.01); also, high seizure frequency was associated with high protein expression in the hippocampus (r = 0.66) [30]. The last study looked at cannabinoid receptors (CBs) 1 and 2, their protein expression, and their induced activation of protein G complexes; the study found less CB1 and CB2 expression (66% and 43% less, respectively) in the hippocampus, which resulted in less potent CB2 receptors [31]. Similarly, a Mexican study looking at protein expression in temporal cortex samples from MDRE patients found a significant increase in the expression of Bcl-2, Caspase-9, and P-glycoprotein (95.2%, 72%, and 72.9%, respectively; *p* < 0.002); no difference was found in the expression of Caspase-3, NT-3, SEMA, or IL1-B [34].

An Argentinian study found a fourfold increase in MDRE risk (OR = 3.88; CI 95% 1.40–10.7; *p* = 0.006) in homozygous patients carrying a 12-repeat polymorphism in the serotonin transporter gene (*SLC6A4*) when compared to carriers of the same 10-repeat gene [25]. Other studies, also from Argentina, looking at individual pediatric MDRE patients found mutations in *KCNT1* (a variant in exon 24) and the overexpression of the *MDR-1* gene, resulting in an increase in P-glycoprotein (P-gp), a known factor in MDRE [26,27,28]. Other studies in Brazil have reported changes in gene expression or polymorphisms in MDRE patients [32,33,35]. One study reported that the T/T genotype at *rs10868235* of the Neurotrophic Tyrosine Kinase Receptor 2 (*NTRK2*) gene was increased in patients with TLE compared to controls with an OR of 1.90 (95%CI 1.17-3.09; *p* = 0.01) [32]. They also showed that patients who were homozygous for the A allele at *rs1443445* showed seizures at younger ages and that the presence of the T allele in a heterozygous or homozygous state was more frequent in patients needing polytherapy (*rs3780645*) with an OR of 4.13 (95%CI 1.68-10.29; *p* = 0.001) [32]. Another study looked at the frequency of the HLA-DRB1*13:02 allele and found that it had significant genetic susceptibility for temporal lobe epilepsy associated with mesial hippocampal sclerosis; however, the significance was lost with Bonferroni correction [33]. Finally, a Brazilian study looking at gene expression using microarrays of RNA expression in samples of hippocampi from temporal lobe MDRE patients found that patients with the familial form of temporal lobe epilepsy had 3109 differently expressed microarray probes and 2274 uniquely expressed genes when compared to controls; conversely, those with the sporadic form had 1716 differently expressed microarray probes and 1077 uniquely expressed genes [35].

### 3.5. Metabolic Causes

A metabolic etiology for MDRE in Latin America was the third most common cause of MDRE, and it includes alterations in metabolites, neuropeptides, and enzymes, altogether representing 15.0% (n = 262) of the total participants included. Most of the included studies in this section come from Brazil.

One study found that MDRE patients have higher levels of malondialdehyde and TROLOX equivalent antioxidant capacity (TEAC) compared to controls [38]. In a similar fashion, other metabolic targets have been identified as being associated with MDRE, albeit less specifically; such markers include an increase in glucose, saturated lipids, isoleucine, proline, and hydroxybutyrate, as found in another study [39]. Nervous-system-related metabolites, including kallikrein 1 (hK1), have been found in the astrocytes of the thalamus, hypothalamus, and frontal cortex. These neuropeptides have two effects in MDRE—glutamate excitotoxicity and increased cortex stimulation caused by hK1 binding to the kinin B2 receptor—resulting in gliosis and chronic inflammation associated with mesial sclerosis and subsequent drug resistance [40]. Also, increased monoamine oxidase A has been associated with a decrease in 5-hydroxytryptamine (5-HT), leading to neuronal excitability, ending up with an increase in the frequency and dominance of bilateral tonic–clonic seizures [41]. On the other hand, a Colombian study of MDR temporal lobe epilepsy participants reported that 57.2% of its participants had a metabolic cause for their epilepsy [15].

### 3.6. Inflammatory Causes

Inflammatory causes of MDRE are the fourth most commonly studied in Latin America, representing 14.8% (n = 258) of all causes.

Different pathways have been discovered regarding the inflammatory genesis of MDRE. A Cuban study demonstrated elevated serum levels of IL-1β and IL-6 before neurosurgery for MDRE treatment compared with the same interleukins post-surgery [17]. IL-1β specifically has a pro-convulsant effect, as reported in a Brazilian study where high levels of this marker were found in MDRE participants; therefore, it might be implicated in MDRE etiology [18]. Additionally, a change in white blood cell parameters has also been reported in MDRE, with an increase in CD8+ cells, CD-25, and HLA-DR in temporal lobe epilepsy [20]. Finally, the metabolic differences between familial and sporadic MTLE have also been studied; an increase in chemokines and cytokine ligands and receptors has been reported, and it shows a direct relationship with the familial form of the disease [19]. Meanwhile, an increase in the epidermal growth factor receptor (EGFR) pathway, phagocytosis, and prostaglandin synthesis appeared to be the associated causes of sporadic cases in [19]. This paper also found an increase in IL-6 and normal serum values of IFN-Y, TNF-a, IL-4, and IL-17 caused by CD3+ and CD4+ T [19].

### 3.7. Infectious Causes

An infectious etiology for MDRE has less information than other causes in Latin America, representing only 8.0% of the total participants analyzed (n = 139). A Brazilian study showed that 67 (10.8%) and 41 (6.6%) participants out of 619 MDRE patients had an infectious etiology (neurocysticercosis and meningitis/encephalitis, respectively) for multidrug resistance [36]. Finally, in Ecuador, a study mentions that 31 MDRE patients had neurocysticercosis and discusses the sub-diagnosis of this disease in the country and other Latin-American countries [37]. It was interesting to find such a small number of papers related to infectious etiologies of MDRE; this is an important research subject and a significant public health gap in Latin-American countries and should be the focus of future efforts in the region. We should determine if infectious etiologies, like neurocysticercosis, result in MDRE infrequently, as reported in other populations, or occur at a higher frequency on our continent.

## 4. Discussion

In our review focusing on the etiology of MDRE in Latin America, we found that structural alterations were the first cause, representing 41.5% of cases, followed by genetic, metabolic, inflammatory, and infectious causes, representing 20.7%, 15.0%, 14.8%, and 8.0% of cases, respectively. Certainly, structural alterations are an important cause of multidrug resistance in Latin America in comparison to Europe, where important etiologic factors have been reported to have both structural and genetic causes [42]. The predisposing polymorphisms in Europe do not seem to influence Latin America; however, inflammatory factors are highly prevalent in both regions. Likewise, infectious and metabolic causes can be linked to the socioeconomic characteristics of the region (less effective public control of metabolic and infectious diseases), which explains why they are not as common in Europe as they are in Latin America [42]. Finally, compared to countries in Asia, structural causes, such as mesial temporal sclerosis, are as common as in Latin America but with differences regarding the frequency of presentation of multidrug resistance in infectious etiologies due to the high prevalence of neurocysticercosis in Latin America [43]

In our study, increases in the expression of P-glycoprotein (P-gp) and the MDR1 gene were associated with MDRE in various studies published in Argentina and Mexico [26,27,28,34]. Certainly, P-gP encoded by the multidrug resistance gene (*MDR1*) belongs to a family of drug transporter proteins [44]. In a Taiwanese study of 118 drug-resistant patients, three specific loci (*C1236T*, *G2677T*, and *C3435T*) in the *MDR1* gene were also found to jointly influence treatment response in epileptic patients [45]. In England, six *3435C>T* polymorphisms in the multidrug transporter gene *ABCB1* were associated with drug resistance [46]. However, in a population of 401 drug-resistant patients in Australia, the CC genotype in the *ABCB1 C3435T* polymorphism was not associated with drug-resistant epilepsy [47], a finding also observed in Ireland [48]; therefore, currently, there is controversy surrounding the role that ABCB1 plays in MDRE, if any. For drug-metabolizing enzymes, whose genetic polymorphisms affect the rate of drug metabolism, a new nonsense variant (c.374G > A) was identified in Pakistan, present only in drug-resistant patients [49]. On the other hand, in Telangana, genetic polymorphisms in *CYP2C92, CYP2C93, CYP2C192*, and *CYP2C193* have shown no significant association with drug-refractory epilepsy [50]. In a similar fashion, our review showed that polymorphisms in the *ABCB1, CYP2C9, SCN1A, CHL1, ABCB1* (variant C3435T), and *ABCC2* (variant *-24C>T*) genes were not associated with MDRE in Latin-American patients [21,22,23,24].

Our review showed that metabolic, enzymatic, and neurophysiological changes such as increased levels of malondialdehyde, TROLOX equivalent antioxidant capacity (TEAC), glucose, saturated lipids, isoleucine, proline, hydroxybutyrate, kallikrein 1, and monoamine–oxidase were associated with MDRE in Latin America [38,39,40,41]. Certainly, metabolic causes of epilepsy have been the focus of research in the last few years, and new metabolic pathways related to the pathogenesis of MDRE have been described. For example, a Chinese study found a relationship between obesity and the systemic inflammatory response that causes MDRE. The study followed 2578 patients, with 345 having an MDRE diagnosis, resulting in an association between obesity and drug resistance due to the generation of a chronic inflammatory environment, the alteration of neurological function, and neurotransmitter anomalies caused by excess weight [51]. In a similar fashion, neuroinflammation has been studied lately as one of the main factors causing epilepsy [52]. Both our review and a study conducted in China show that pro-inflammatory molecules like IL-1B and IL-6 are increased in refractory epileptic patients due to microglial and astrocytic activation [52]. However, inflammation cannot occur spontaneously; it has various causes such as endogenous and exogenous triggers, homeostasis disruption, infections, and gene expression variation, among others [52]. An Italian study suggests that the major causes of inflammation are pathologic infections caused by viruses, which result in encephalitis [53]. The mechanism suggested by this study is a reduction in the epileptogenic threshold caused by an increase in pro-inflammatory molecules [53]. Certainly, completely separating inflammation and infection as causative agents of MDRE is difficult, as they are interconnected. For example, an Indian retrospective study found an association between MDRE and neurocysticercosis caused by inflammation and morphologic effects generated by cysticerci, leading to gliosis, axonal, and synaptic affectation, highlighting the importance of an early diagnosis and treatment to prevent complications such as MDRE [54,55]. Certainly, neuroglia has been proven to play an important role in inadequate inflammatory responses in the nervous system; reactive astrocytosis can result in epileptogenic genesis, and miscommunication between glial cells can result in more reactive astrocytosis, worsening inflammation and lowering the seizure threshold of the affected brain tissue [56].

### Study Limitations

To contextualize the data obtained from this systematic review, some limitations must be acknowledged. Firstly, this study is exclusively focused on Latin-American countries. Although this specificity enhances the investigation, it concurrently restricts the information available regarding the causes of refractory epilepsy, as other countries conduct more extensive research on this subject. Secondly, research in Latin America has revealed various constraints in data retrieval attributable to the healthcare systems of individual countries. There are patients who have been misdiagnosed as not having epilepsy and not having MDRE. Public health in various nations suffers from a deficiency of diagnostic instruments and treatment alternatives, which constrains the identification of epilepsy etiology and patient outcomes; this might have resulted in an underestimation of MDRE etiologies. Thirdly, multidrug treatment might be challenging to implement due to socioeconomic constraints and the availability of medications in each nation, resulting in a poor response to MDRE. An additional critical factor to consider is the restricted patient follow-up resulting from voluntary patient attrition, challenges in accessing remote regions, and socioeconomic deprivation. Also, misdiagnoses can frequently occur due to restricted access to sophisticated diagnostic instruments and insufficient training for healthcare professionals. Consequently, patients may be erroneously classified as having multidrug-resistant epilepsy when their disease could be amenable to alternative treatments or attributable to distinct underlying factors. Not all studies indicated the utilization of the ILAE criteria for patient enrollment. Finally, the ultimate constraint of our investigation is language. The languages selected for information gathering were solely English and Spanish. We could not include Portuguese, an important language in Latin America, and this could have resulted in the involuntary exclusion of relevant articles.

## 5. Conclusions

Our systematic review highlights the multifactorial nature of multidrug-resistant epilepsy (MDRE) in Latin America, with structural abnormalities (particularly hippocampal sclerosis) emerging as the most common etiology, followed by genetic, metabolic, inflammatory, and infectious causes. The high burden of structural and acquired etiologies underscores the critical role of socioeconomic and healthcare disparities in the pathogenesis of MDRE within the region. Unlike findings from high-income countries, Latin-American populations appear less influenced by common pharmacogenetic polymorphisms, reinforcing the importance of region-specific genomic and environmental studies. Our findings call attention to a significant underrepresentation of infectious etiologies in the literature, despite neurocysticercosis and other endemic infections being well-known contributors to epilepsy in Latin America. This evidences a stark research and public health gap that must be addressed through targeted epidemiological surveillance, improved access to diagnostics, and regionally tailored treatment protocols. Moreover, the convergence of metabolic and inflammatory processes in the pathophysiology of MDRE demands a re-evaluation of therapeutic approaches, shifting from purely pharmacological strategies to a more integrated model that addresses immunometabolic dysfunction. The growing evidence of glial dysfunction and pro-inflammatory cascades, particularly the roles of IL-1β, IL-6, and reactive astrocytosis, suggests that targeting neuroinflammation may represent a novel avenue for intervention in refractory cases. In conclusion, MDRE in Latin America cannot be understood through a singular lens. It is a product of complex interplays between biology, the environment, and health system inequities. Future research must prioritize prospective, multicentric, and molecularly informed studies that include underserved populations, investigate novel biomarkers, and assess emerging therapies beyond antiepileptic drugs, ultimately aiming to reduce the burden of MDRE in one of the world’s most vulnerable regions.

## Figures and Tables

**Table 1:** Risk of bias.

Author (Year)	Study Type	Level of Bias
Aberastury, M. (2016) [5]	Cross-sectional	High
Alves, S. (2013) [6]	Cohort	High
Bilevicius E. (2010) [7]	Case–control	Moderately low
Calderon-Ospina, C. (2020) [21]	Cohort	Moderately low
Calderón-Toledo, S. (2019) [22]	Case–control	Moderately low
de Araújo Filho GM. (2018) [38]	Cross-sectional	Moderately low
Duarte, J. (2018) [8]	Cohort	High
Garcia (2017) [9]	Cohort	High
Godoi AB. (2022) [39]	Cross-sectional	High
Horta WG. (2015) [33]	Cross-sectional	High
Kauffman MA. (2009) [25]	Cross-sectional	Moderately low
Kobayashi (2001) [10]	Case–control	Moderately low
Kravetz (2021) [26]	Case report	Moderately low
Ladino (2015) [11]	Case report	Moderately low
Lara-Giron (2021) [12]	Case report	Moderately low
Latini (2022) [13]	Clinical trial	Moderately low
Lazarowski (2004) [27]	Case report	Moderately low
Lazarowski (1999) [28]	Case report	Moderately low
Lopez-Garcia (2017) [23]	Cross-sectional	Moderately low
Lorigados-Pedre (2018) [17]	Case–control	Moderately low
Lorigados-Pedre (2004) [20]	Cross-sectional	Moderately low
Maradei-Anaya (2013) [24]	Cross-sectional	Moderately low
Martinez-Levy (2016) [29]	Case–control	Moderately low
Maurer-Morelli (2022) [35]	Case–control	Moderately low
Navarrete-Modesto (2019) [30]	Case–control	Moderately low
Nunez-Lumbreras (2020) [31]	Case–control	Moderately low
Pereira, M. (2022) [36]	Cohort	Moderately low
Pinheiro, A. (2012) [14]	Cross-sectional	Moderately low
Placencia, M. (1994) [37]	Case–control	Moderately low
Quiroga, P. (2022) [15]	Cohort	Moderately low
Rosa, D. (2016) [19]	Case–control	Moderately low
Sanabria C. (2016) [16]	Cross-sectional	Moderately low
Santos, R. (2021) [18]	Case–control	Moderately low
Simões, P. (2011) [40]	Case–control	Moderately low
Torres, C. (2017) [32]	Case–control	Moderately low
Vega-García, A. (2021) [34]	Case–control	Moderately low
Vincentiis, S. (2019) [41]	Case–control	Moderately low

## Data Availability

All data are available in the manuscript.

## Supplementary Materials

The following supporting information can be downloaded at: https://www.mdpi.com/article/10.3390/biom15040576/s1, Supplementary File: Complete search criteria used in each database.

## Abbreviations

The following abbreviations are used in this manuscript:

ABS	ATP-Binding Cassette
ASM	Anti-seizure medication
BDNF	Brain-derived neurotrophic factor
CB	Cannabinoid receptor
CYP	Cytochrome
hK1	Kallikrein 1
ILAE	International League Against Epilepsy
MDRE	Multidrug-resistant epilepsy
MRI	Magnetic resonance imaging
MTLE	Mesial Temporal Lobe Epilepsy
NHLBI	National Heart, Lung, and Blood Institute
NTRK2	Neurotrophic Tyrosine Kinase Receptor 2
PRISMA	Preferred Reporting Items for Systematic Reviews and Meta-Analysis
P-gp	P-glycoprotein
SLC	Serotonin transporter gene
SNP	Single-nucleotide polymorphism
TEAC	TROLOX equivalent antioxidant capacity
TLE	Temporal lobe epilepsy
WOS	Web of Science
5-HT	5-hydroxytryptamine
