# Supplementary material for: Etiologies of Multidrug-Resistant Epilepsy in Latin America: A Comprehensive Review of Structural, Genetic, Metabolic, Inflammatory, and Infectious Origins: A Systematic Review
Source: Biomolecules. 2025 Apr 12;15(4):576. doi: 10.3390/biom15040576 (PMC12025188; doi:10.3390/biom15040576)
Supplement: Supplementary file 1 [file biomolecules-15-00576-s001.zip › biomolecules-3571630-supplementary.pdf]

Supplementary Materials

Search strategy for each database:

PubMed:

((Epileps* OR (Epileps*, Cryptogenic) OR (Cryptogenic Epileps*) OR (“Epilepsy”[MESH])) AND ((Pharmacoresistant epilepsy) OR (drug-resistant epilepsy) OR (Epileps*, Drug Resistant) OR (Drug Resistant Epileps*) OR (Resistant Epileps*, Drug) OR (Medication Resistant Epileps*) OR (Epileps*, Medication Resistant) OR (Resistant Epileps*, Medication) OR (Medication Resistant Epileps*) OR (Epileps*, Medication Resistant) OR (Refractory Epileps*) OR (Epileps*, Refractory) OR (Drug Refractory Epileps*) OR (Refractory Epileps*, Drug) OR (“Drug Resistant Epilepsy”[MESH])) AND ((“Latin America”[MESH]) OR (“South America”[MESH]) OR (“Central America”[MESH]) OR (Argentina) OR (Bolivia) OR (Brazil) OR (Chile) OR (Colombia) OR (Costa Rica) OR (Cuba) OR (Ecuador) OR (El Salvador) OR (Guyana) OR (Guatemala) OR (Haiti) OR (Honduras) OR (Jamaica) OR (Mexico) OR (Nicaragua) OR (Paraguay) OR (Panama) OR (Peru) OR (Puerto Rico) OR (Dominican Republic) OR (Surinam) OR (Uruguay) OR (Venezuela))

Scopus:

TITLE-ABS-KEY (((pharmacoresistant AND epilepsy) OR (drug-resistant AND epilepsy) OR (epilepsy, AND drug AND resistant) OR (drug AND resistant AND epilepsy) OR (resistant AND epilepsy, AND drug) OR (medication AND resistant AND epilepsy) OR (epilepsy, AND medication AND resistant) OR (resistant AND epilepsy, AND medication) OR (medication AND resistant AND epilepsy) OR (epilepsy, AND medication AND resistant) OR (refractory AND epilepsy) OR (epilepsy, AND refractory) OR (drug AND refractory AND epilepsy) OR (refractory AND epilepsy, AND drug) OR (drug AND resistant AND epilepsy)) AND ((latin AND america) OR (south AND america) OR (central AND america) OR argentina OR bolivia OR brazil OR chile OR colombia OR (costa AND rica) OR cuba OR ecuador OR (el AND salvador) OR guyana OR guatemala OR haiti OR honduras OR jamaica OR mexico OR nicaragua OR paraguay OR panama OR peru OR (puerto AND rico) OR (dominican AND republic) OR surinam OR uruguay OR venezuela))

Web of Science:

((Pharmacoresistant epilepsy) OR (drug-resistant epilepsy) OR (Epilepsy Drug Resistant) OR (Drug Resistant Epilepsy) OR (Medication Resistant Epilepsy) OR (Epilepsy Medication Resistant) OR (Medication Resistant Epilepsy) OR (Epilepsy Medication Resistant) OR (Refractory Epilepsy) OR (Drug Refractory Epilepsy) OR (Drug Resistant Epilepsy)) AND ((Latin America) OR (South America) OR (Central America) OR Argentina OR Bolivia OR Brazil OR Chile OR Colombia OR (Costa Rica) OR Cuba OR Ecuador OR (El Salvador) OR Guyana OR Guatemala OR Haiti OR Honduras OR Jamaica OR Mexico OR Nicaragua OR Paraguay OR Panama OR Peru OR (Puerto Rico) OR (Dominican Republic) OR Surinam OR Uruguay OR Venezuela)
